# Ventilation

**Published:** 1899-04-01

**Authors:** Alice Ravenhill


### The Institutional Workshop.

### "VENTILATION."

By Alice Ravenhill.

The primary importance of the subject of ventilation is widely recognised by all interested in promoting the health of the community, whether considering the matter from a preventive or remedial standpoint. To secure the efficient ventilation of large buildings in this variable climate, by natural means, offers a problem beset with intricacies, mere change of air being far from the only essential requirement—a suitable temperature, humidity of atmosphere, and freedom from draughts should also be secured. As a consequence, mechanical, rather than natural means of ventilation are increasingly resorted to in public institutions, schools, &c., though some diversity of opinion exists as to the respective merits of the two methods. Most of the Board schools in London, for instance, are ventilated by natural means, whereas in large provincial towns some system of mechanical ventilation has frequently been adopted, and figures are available to prove the resultant advantages to the scholars.

One of the largest installations laid down in England for mechanical ventilation is that on the Plenum system at the new General Hospital at Birmingham, opened about eighteen months ago. The internal capacity of this great hospital is two million cubic feet, and provision is made for propelling mechanically (by means of an ingenious arrangement of inlet shafts for air previously purified and warmed or cooled according to the external temperature, and of extract shafts for the removal of the vitiated atmosphere) a total of twenty million cubic feet of air throughout the building every hour, continuously, night and day. To ensure the successful application of the system the windows are hermetically sealed, and serve for lighting purposes only, while fireplaces are rendered unnecessary and are therefore non-existent, the only open grate in the building being that in the house governor's room.

The great cost of such installations as this at Birmingham, and the natural desire to learn whether results justify the outlay involved, lead to the working of the Plenum system in large institutions being keenly watched by both medical and sanitary authorities, and an opportunity of personal inspection of schools, asylums, or hospitals, so ventilated, is acceptable to anyone interested in the important problem of securing purity of atmosphere either for the sick or sound. Those sceptical as to its advantages bring forward instances of its uncertain working—one installation proving a success, another nominally on identical lines verging on the reverse. The supporters, however, point with satisfaction to the results of four years' experience in the Victoria Infirmary, Glasgow, which show that the working expenses for heating, ventilation, and cost of electric lighting about equal those previously incurred for gas and coals for fires, the patients being meanwhile supplied with 1,000 cubic feet of air every ten minutes; but it is not considered that sufficient data yet exist to furnish comparative statistics as to length of residence, death-rate, &c., though it is believed these would prove to be slightly less than in the Royal and Western Infirmaries in the same city.

Some years also must necessarily elapse before sufficient material will be available from which to draw accurate conclusions or compile statistics on these important points at Birmingham, but the publication of the impression received on visiting the new General Hospital may perhaps serve to elicit expressions of opinion on this system of ventilation from those who have had more or less extensive practical experience of working in institutions where it is in force. The visit in question was paid with an open mind, and in conjunction with others anxious to observe in practice a previously carefully studied theory.

As amply exemplifying the force of life-long habit (which, in this case, evidently associates change of air with more or less sensation of draught), the point most generally commented upon was the apparently unnatural stillness of the atmosphere, whereas actually the air was being much more rapidly changed than is usual, or possible, by natural means; the perfect adjustment of all the essential factors resulting in an entire absence of draught. It seems that this subjective sensation is almost invariably experienced by new comers to the hospital, though both nurses and patients gradually become accustomed to what scientists assert should be a natural (but which to most of us at present appears to be a somewhat artificial) atmosphere. It has been stated that this feeling of oppression does not meet with the entire approval of the visiting staff, as exercising upon them an indefinably depressing effect. The atmosphere of those wards visited struck the party as somewhat close, lacking in freshness; this impression being attributed not to any defect in the purity of the air, but to the want of sufficiently rapid power of accommodation to new conditions on the part of the visitor! Indeed, it is considered that as a result of the effectual, but imperceptible, change of air, no risk can accrue from nursing six or seven cases of enteric fever, and nearly as many of pneumonia and phthisis in one ward, the experience at the Glasgow Victoria Infirmary showing that pneumonia and phthisis cases do exceptionally well in wards ventilated on this system.

The excellent lavatory arrangements at Birmingham are isolated in corner turrets, separated from the wards by two sets of doors, divided in their turn by a few feet of passage. These doors were originally so heavy that it was found necessary to divide them down the middle and rehang them as two; their weight being too great for one woman to push open unassisted; the short passages have cross lights, but ventilating shafts are believed to be unnecessary. The question presented itself to one of the medical members of the party as to whether, under certain possible conditions, there might not be undesirable communication between the air of the lavatories and that in the wards. For instance, all "typhoid" linen is collected into galvanised pails in the lavatories and removed twice daily by a porter, instead of being sent down the shaft in the corridor with the other soiled linen; the omission to carefully close the lids of these pails—not, perhaps, unlikely to occur occasionally—might not then be unattended by risk during the few hours their contents remain in the lavatories, were the intermediate doors ever propped open during the rush of ward work.

The kitchens on the top floor of this fine hospital are alone well worth a visit to those interested in labour-saving appliances, but again, the first impression experienced was a craving for the familiar, but forbidden, draught. The regiment of substantial oak dinner wagons was exhibited with much pride, ranged against the hot-water plate in readiness for the next day's meal, and the hermetically closing lids were raised to show the beef tea and milk cans, pudding pans, &c., ranged in readiness for to-morrow's supplies; but while admiring this desirable provision for the comfort of the patients, some of the party confessed, with shame and confusion of face, their preference for the old-fashioned system which preached the free exposure to the outside air of all vessels used in connection with food, rather than their bestowal in closed chests or in a confined atmosphere.

Where the initial outlay on hospital construction is so compulsorily a matter of supreme importance it would seem advisable to secure very convincing proofs of the advantages derived from the mechanical ventilation in force at Glasgow and Birmingham before incurring the cost of installing the Plenum system in other hospitals. It must be borne in mind that with an allowance of 1,000 cubic feet of air space per bed adequate ventilation by natural means is practically always possible, any slight or temporary difficulties being more than counterbalanced in the eyes of certain sanitary experts by the advantages derived from open grates, and the possibility of warming by radiant versus convected heat. At Birmingham the allowance of air space per bed is 1,800 cubic feet, so that these difficulties would be reduced to almost vanishing point. On the other hand, mechanical ventilation seems a necessity in the case of elementary schools, with an air space of only 100 to 140 cubic feet per head; but the experience gained by visiting several schools where the Plenum system is installed tends even then to the conviction that complete freshness is only assured where mechanical methods of changing the air can be supplemented by the "natural" opening of windows and doors during the recreation intervals, permitting thorough flushing of class room and hall, otherwise a stale, stuffy smell is usually unpleasantly perceptible to sensitive nostrils. Doubtless allowance must be made in most cases for imperfect application of the principles involved; outlets may be unsatisfactorily arranged, fans may be overworked, or the system may suffer from efforts to minimise expense, and, notably, by placing it in the charge of ignorant attendants.

Depending, as it does, on highly skilled management for its efficiency, some scientific training would seem desirable for its controllers. For instance, if comment be made to those in charge as to the closeness of school-room or ward no such confusion of ideas should exist as to cause the temperature to be quoted in proof of the satisfactory condition of the air. So long as but one person in charge of a part of any building thus ventilated fails to recognise that thermometer registers temperature but does not gauge atmospheric impurities, so long will an element of possible danger to health exist for those dependent on this and kindred means of air-purification. The modest outlay necessary to supply all schools and public institutions with Dr. Scurfield's Ventilation Indicator would be abundantly justified if, in addition to its value in quickly estimating the efficiency of the ventilation of a room, it might serve as the basis for practical object lessons to those responsible for the same as to the distinction between warm air and "stuffy," and this distinction promises to need forcible emphasis until the serious study of practical hygiene takes a more important place in the training curriculum of teachers, nurses, and caretakers.

In conclusion, the moral consequence of seeing only closed windows upon a large body of children or of ignorant patients seem worth consideration, susceptible as is this objection to being dismissed as purely sentimental. Nevertheless, after the first novelty has worn off and the subject been talked threadbare to the few, it is hardly probable that the many successive series of patients will be enlightened by their ever busy attendants as to the reasons why the windows are used only to admit light, or that overworked nurses will enter into an explanation of the elaborate construction of walls and shafts for the admission and extraction of air, and dilate upon the ingenious mechanism ever at work to propel and purify the incoming atmosphere. Until, too, the instruction of scholars in the elements of sanitation is held in far higher estimation among the rank and file of school-teachers than is now the case, a minority only of the pupils will grasp the reason why windows are made to admit light only at school, but must be depended on to furnish both air and light at home. One argument advanced in favour of erecting handsome, well-equipped Board schools is the hope that the children may be incited to reproduce in their homes the surroundings with which they are familiarised at school. It is conceivable that in respect of unopened windows this argument may receive a wealth of support.

